# From Psychosis to Recovery: A Case Report of N-Methyl-D-Aspartate (NMDA) Receptor Encephalitis

**DOI:** 10.7759/cureus.67272

**Published:** 2024-08-20

**Authors:** Sava Nanda Gopal, Deepthi Vakati, Saranya Palanisamy, Kanimozhi David, Kannan Rajendran

**Affiliations:** 1 Internal Medicine, Saveetha Medical College and Hospitals, Saveetha Institute of Medical and Technical Sciences, Saveetha University, Chennai, IND

**Keywords:** immunotherapy, antibodies, encephalitis, nmda receptor, autoimmune

## Abstract

Autoimmune N-methyl-D-aspartate (NMDA) receptor encephalitis is an increasingly recognized cause of severe neuropsychiatric illness, particularly in young individuals. This case report presents a detailed account of a patient diagnosed with NMDA receptor encephalitis, highlighting the clinical presentation, diagnostic challenges, and treatment approach. The patient exhibited initial symptoms of psychosis and memory disturbances, which rapidly progressed to seizures and autonomic instability, reflecting the characteristic progression of the disorder.

## Introduction

Autoimmune N-methyl-D-aspartate (NMDA) receptor encephalitis (ANRE) is a rapidly progressive and potentially life-threatening neurological disorder, first described in 2007 by Dalmau et al. [1]. It is characterized by the presence of autoantibodies against the NR1 subunit of NMDA receptors, leading to profound neuropsychiatric symptoms. Although it predominantly affects young women, ANRE can occur across all age groups and genders, presenting a significant challenge in neuroimmunology and neuropsychiatry [2,3].

The clinical presentation of ANRE is often dramatic, with patients experiencing a wide array of symptoms, including psychosis, memory deficits, seizures, autonomic instability, and movement disorders [4]. This heterogeneity in clinical manifestations can lead to diagnostic challenges and frequent misdiagnoses, often as primary psychiatric disorders or infectious encephalitis [5]. Early recognition and treatment are crucial for improving outcomes, underscoring the importance of heightened awareness among healthcare professionals.

Despite advances in understanding its immunopathogenesis, clinical features, and treatment strategies, many aspects of ANRE remain incompletely understood. This review aims to provide a concise overview of the current knowledge of ANRE, including its epidemiology, pathophysiology, clinical presentation, diagnostic criteria, and treatment options, while highlighting areas for future research.

## Case presentation

An 18-year-old female student from Southern India presented to our hospital with a sudden onset of behavioral changes over seven days. The patient exhibited decreased interaction with family members, loss of interest in her surroundings, and eventually developed new-onset seizures followed by postictal confusion. Initially she was brought to the psychiatry department, but due to the progression of her symptoms, she was referred to the neurology department to rule out an organic cause.

Clinical examination

On examination, the patient was apathetic, displaying catatonia, stereotypic movements, orofacial dyskinesia, mutism, and psychosis. Motor examination revealed normal muscle power, but all deep tendon reflexes were exaggerated, and plantar reflexes were bilaterally flexor. Sensory and cranial nerve examinations were within normal limits, and her vital signs were stable.

Lab investigations

Laboratory tests revealed normocytic normochromic anemia with a hemoglobin level of 11 g/dL (normal range for females: 12-15.5 g/dL). The total leukocyte count was 8,600 cells/µL (normal range: 4,000-11,000 cells/µL) and platelets were 371,000 cells/µL (normal range: 150,000-450,000 cells/µL). Liver and renal function tests were within normal limits.

Cerebrospinal fluid (CSF) analysis showed a mildly elevated total cell count, and tests for the herpes simplex virus (HSV-1 and HSV-2) were negative, as summarized in Table 1.

**Table 1:** CSF analysis. normal range: indicates the typical range of values for a healthy individual; mildly elevated: slightly above the normal range, potentially indicating a minor issue; negative: no presence of the tested element (e.g., no infection, no malignant cells); weakly positive: a low level of the tested element, suggesting a possible but not definitive presence (e.g., possible autoimmune condition). CSF: cerebrospinal fluid; ADA: adenosine deaminase; TB: tuberculosis; HSV: herpes simplex virus; NMDA: N-methyl-D-aspartate; PCR: polymerase chain reaction; AFB: acid-fast bacilli

Test	Result	Implication
CSF Total Cell Count	7 cells/µL	Mildly elevated; normal is usually 0-5 cells/µL. Mild pleocytosis, which may indicate mild inflammation or infection.
Differential Count (DC)	0	No specific differential count provided.
Malignancy	Negative	No evidence of malignant cells.
CSF-Acid-Fast Bacilli	Negative	No evidence of tuberculosis.
Gram Stain	Negative	No bacterial infection detected.
CBNAAT	Negative	No evidence of tuberculosis (confirming CSF-AFB results).
CSF Adenosine Deaminase (ADA)	1.8 U/L	Low; not suggestive of tuberculosis (TB meningitis often has higher ADA levels).
CSF Protein	47 mg/dL	Normal range (15-45 mg/dL); mild elevation could indicate inflammation.
CSF Glucose	86 mg/dL	Normal range; typically two-thirds of blood glucose levels.
CSF Culture	No growth	No bacterial growth, indicating no bacterial infection.
CSF Encephalitis Panel	Weakly positive for NMDA	Suggests possible autoimmune encephalitis, particularly anti-NMDA receptor encephalitis.
CSF HSV-1	0.05	No evidence of herpes simplex virus infection.
CSF HSV-2	0.04	No evidence of herpes simplex virus infection.
CSF PCR	Negative	No evidence of viral or bacterial DNA/RNA, ruling out specific infections tested by PCR.

**Figure 1:**
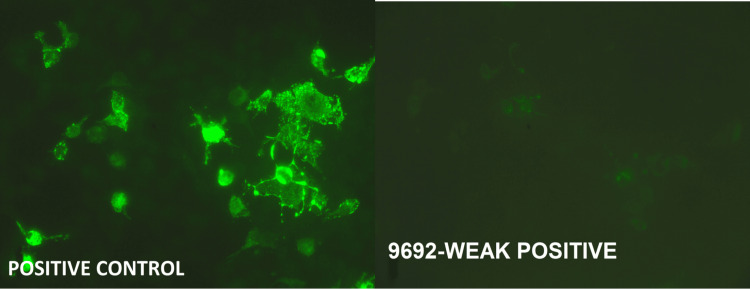
NMDA receptor sensitivity. Positive control is a sample known to contain the target antigen (in this case, NMDA receptors) and should exhibit a strong positive reaction. It is used to validate the test's accuracy and ensure that the test conditions are functioning properly. Weak positive indicates a low level of target antigens (NMDA receptors) in the sample. The fluorescence is present, but not as intense as in a strong positive or control sample, suggesting a lower concentration of the antigen. NMDA: N-methyl-D-aspartate

The autoimmune antibody panel of the CSF showed a weakly positive result for NMDA receptor antibodies as illustrated in Figure 1.

The antinuclear antibody (ANA) profile, conducted to screen for autoantibodies revealed no significant autoantibodies as shown in Table 2.

**Table 2:** ANA profile. negative: no significant level of autoantibodies detected; borderline (B): autoantibody level is at the threshold, not clearly negative or positive. ANA: antinuclear antibody

ANA	Result
Mi-2	Negative
Ku	Negative
RNP/Sm (RNP)	Borderline (B)
Sm	Negative
SS-A native (60kDa) (SSA)	Negative
Ro-52 recombinant (Ro 52)	Negative
SS-B (SSB)	Negative
Scl-70 (Scl)	Negative
PM-Scl100 (PM100)	Negative
Jo-1 (Jo)	Negative
Centromere B (CB)	Negative
PCNA	Negative
dsDNA (DNA)	Borderline (B)
Nucleosomes (NUC)	Negative
Histone (HI)	Negative
Ribosomal Protein (RIB)	Negative
AMA-M2	Borderline (B)
DFS70	Negative

**Figure 2:**
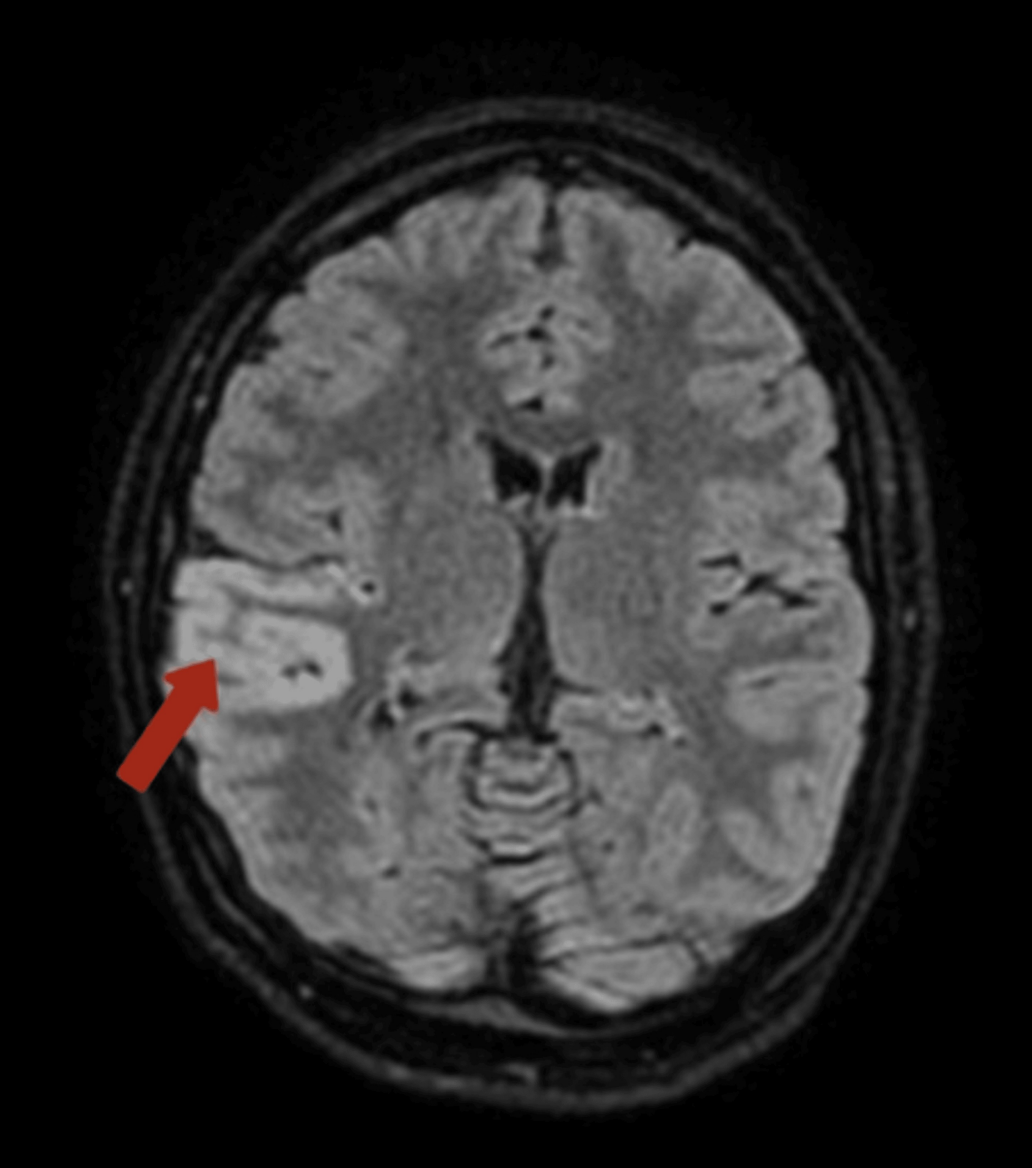
The MRI image-T2 FLAIR displays hyperintensities in the cortical and subcortical regions of the right temporo-parietal lobe. The hyperintensities observed on T2 FLAIR sequences are indicative of pathological changes, which may include edema, demyelination, gliosis, or other abnormalities. FLAIR: fluid attenuated inversion recovery

**Figure 3:**
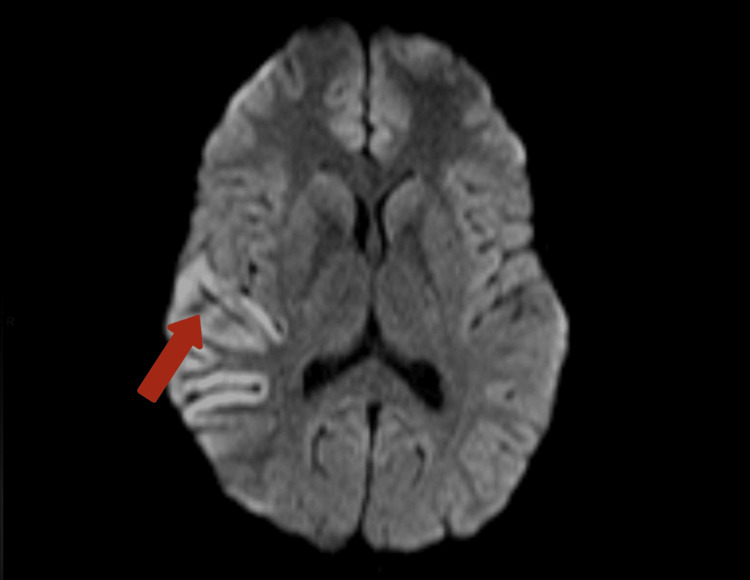
MRI image-DWI showing fairly defined gyriform areas of DWI hyperintensities with corresponding subtle ADC hypointensities. This is an MRI-T2 weighted image indicating an area of hyperintensity in the cortical region of the right-temporo parietal lobe. DWI: diffusion-weighted imaging; ADC: apparent diffusion coefficient

**Figure 4:**
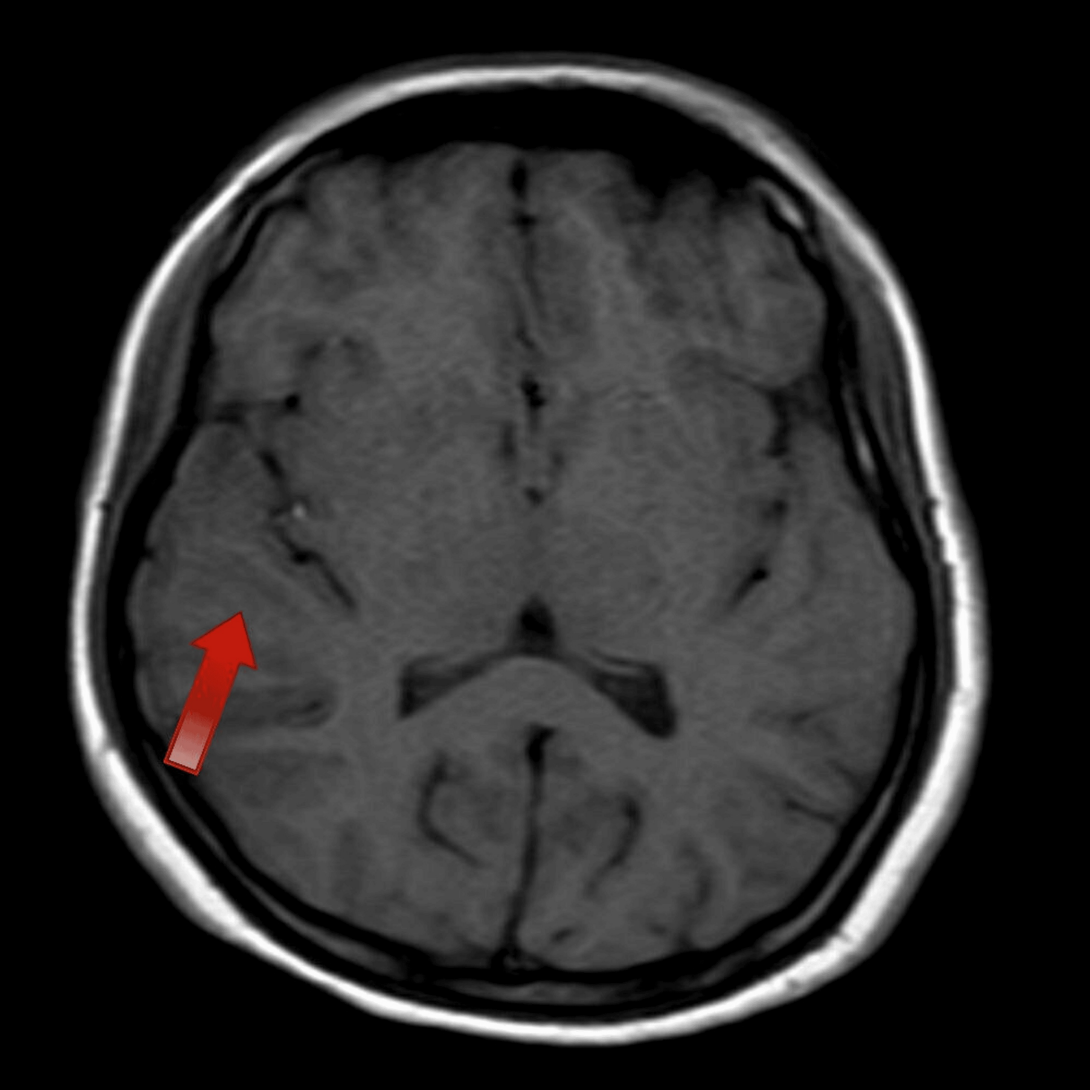
MRI image-T1 showing hypointense. This MRI image obtained using the T1-weighted sequence displays hypointense regions (areas appearing darker) within the brain.

MRI of the brain revealed focal cortical edema with mild gyriform areas of diffusion restriction involving the right temporo-parietal lobe, suggestive of encephalitis potentially of an autoimmune or infectious origin. This is depicted in Figures 2, 3, 4.

Treatment

Upon confirming the diagnosis, the patient underwent three cycles of plasmapheresis. Following treatment, her general condition improved significantly, and she became responsive to oral commands; her orofacial dyskinesia subsided, and she regained awareness of her surroundings. The patient was initially treated with intravenous steroids, which were later transitioned to oral steroids along with other supportive medications. She was subsequently discharged with instructions for follow-up.

## Discussion

The heterogeneity in clinical manifestations can pose diagnostic challenges, often resulting in initial misdiagnoses such as primary psychiatric disorders or infectious encephalitis [5]. Early recognition and prompt treatment are essential for improving patient outcomes, highlighting the need for increased awareness and understanding of this disorder among healthcare professionals. Recent advancements in the understanding of ANRE have shed light on the underlying immunopathogenesis, clinical features, diagnostic approaches, and therapeutic strategies [6]. Despite these advancements, many questions remain unanswered, and ongoing research is crucial for unraveling the complexities of this disease. This review aims to provide a comprehensive overview of the current knowledge on ANRE, discussing its clinical presentation, treatment, and future directions in research. Increased awareness and understanding of ANRE are imperative for prompt diagnosis and effective management [7]. Diagnostic approaches have evolved, incorporating both clinical assessment and advanced laboratory techniques to detect specific autoantibodies. Therapeutic strategies have also progressed, with immunotherapy playing a central role in management along with supportive care tailored to the patient's specific symptoms [8].

Despite these strides, numerous aspects of ANRE remain under investigation. The complexity of its pathophysiology and the variability in clinical outcomes highlight the need for continued research. This review seeks to provide a thorough examination of current knowledge on ANRE, addressing its epidemiology, pathophysiology, clinical presentation, diagnostic criteria, and treatment options. Furthermore, it aims to explore future research directions that may offer deeper insights into this multifaceted disorder. The estimated incidence of ANRE is approximately 1.5 per million people per year, highlighting its rarity in the general population [8]. Despite its low incidence, the disorder is of significant clinical interest due to its unique presentation and the dramatic response to immunotherapy in many cases.

The disorder exhibits a higher prevalence in females, particularly those between the ages of 18 and 45. This gender predilection is evident in various studies and case series that report a female-to-male ratio of approximately 4:1 [9]. This higher incidence in young women is thought to be related in part to the association with ovarian teratomas, which are known to express NMDA receptor antigens that can trigger the autoimmune response. However, ANRE can occur in males and individuals of all ages, from infants to the elderly, indicating that while gender and age are significant factors, they are not exclusive determinants.

The association between ANRE and tumors, particularly ovarian teratomas, is well documented and underscores the paraneoplastic nature of the disorder in many cases. Approximately 50% of young female patients with ANRE have an underlying ovarian teratoma, which suggests a strong link between tumor presence and disease pathogenesis [10]. These tumors express NMDA receptors, which likely serve as the initial antigenic target, leading to an autoimmune response against these receptors in the brain. This hypothesis is supported by the rapid improvement observed in many patients following tumor removal and immunotherapy.

The pathophysiology of anti-NMDA receptor encephalitis (ANRE) centers around the production of autoantibodies against the NR1 subunit of N-methyl-D-aspartate (NMDA) receptors. These receptors are integral to synaptic transmission and plasticity in the central nervous system, playing a critical role in cognitive functions, memory, and learning [11]. The presence of these autoantibodies leads to a cascade of pathological events that culminate in significant neurological impairment.

When autoantibodies bind to the NR1 subunit of NMDA receptors they cause the receptors to be internalized and degraded. This process results in a marked decrease in the surface expression of NMDA receptors on neurons, leading to impaired synaptic function and neurotransmission. The loss of NMDA receptor activity disrupts excitatory neurotransmission and synaptic plasticity, which are essential for normal cognitive and motor functions [12].

The treatment of ANRE involves immunotherapy and supportive care. First-line therapies include corticosteroids, intravenous immunoglobulin (IVIG), and plasmapheresis. For patients who do not respond to initial treatment, second-line therapies such as rituximab and cyclophosphamide may be considered. Tumor removal is crucial in paraneoplastic cases, particularly those associated with ovarian teratomas. Early and aggressive treatment is associated with better outcomes, although some patients may experience relapses and require long-term immunosuppression [7].

Future research in ANRE should focus on identifying the precise mechanisms underlying autoantibody production and the role of genetic and environmental factors in disease susceptibility. Improved diagnostic tools and biomarkers are needed for early and accurate diagnosis. Additionally, the development of targeted therapies that can modulate the immune response without compromising overall immune function is a critical area of investigation. Long-term studies are necessary to understand the chronic sequelae of ANRE and optimize management strategies for preventing relapses [3].

## Conclusions

Autoimmune NMDA receptor encephalitis is a complex and severe neurological disorder that requires prompt recognition and treatment. Advances in understanding the immunopathogenesis and clinical features of ANRE have significantly improved diagnostic and therapeutic approaches. However, many aspects of the disease remain poorly understood, necessitating ongoing research. Enhanced awareness and knowledge of ANRE among healthcare providers are essential for improving patient outcomes and advancing the field of neuroimmunology.
